# Circulating Leukocytes and Oxidative Stress in Cardiovascular Diseases: A State of the Art

**DOI:** 10.1155/2019/2650429

**Published:** 2019-10-15

**Authors:** Speranza Rubattu, Maurizio Forte, Salvatore Raffa

**Affiliations:** ^1^Department of Clinical and Molecular Medicine, School of Medicine and Psychology, Sapienza University of Rome, Italy; ^2^IRCCS Neuromed, Pozzilli (Isernia), Italy; ^3^Ultrastructural Pathology Lab-Medical Genetics and Advanced Cellular Diagnostics Unit, Sant'Andrea University Hospital, Rome, Italy

## Abstract

Increased oxidative stress from both mitochondrial and cytosolic sources contributes to the development and the progression of cardiovascular diseases (CVDs), and it is a target of therapeutic interventions. The numerous efforts made over the last decades in order to develop tools able to monitor the oxidative stress level in patients affected by CVDs rely on the need to gain information on the disease state. However, this goal has not been satisfactorily accomplished until now. Among others, the isolation of circulating leukocytes to measure their oxidant level offers a valid, noninvasive challenge that has been tested in few pathological contexts, including hypertension, atherosclerosis and its clinical manifestations, and heart failure. Since leukocytes circulate in the blood stream, it is expected that they might reflect quite closely both systemic and cardiovascular oxidative stress and provide useful information on the pathological condition. The results of the studies discussed in the present review article are promising. They highlight the importance of measuring oxidative stress level in circulating mononuclear cells in different CVDs with a consistent correlation between degree of oxidative stress and severity of CVD and of its complications. Importantly, they also point to a double role of leukocytes, both as a marker of disease condition and as a direct contributor to disease progression. Finally, they show that the oxidative stress level of leukocytes reflects the impact of therapeutic interventions. It is likely that the isolation of leukocytes and the measurement of oxidative stress, once adequately developed, may represent an eligible tool for both research and clinical purposes to monitor the role of oxidative stress on the promotion and progression of CVDs, as well as the impact of therapies.

## 1. Introduction

Oxidative stress is the product of several intracellular sources such as mitochondrial electron transport chain (ETC), nicotinamide adenine dinucleotide phosphate oxidase (NADPH), nitric oxide synthase, and xanthine oxidase [1–4]. Several antioxidant mechanisms also exist within the mitochondrial compartment (uncoupling proteins, thioredoxins, glutathione peroxidase, and superoxide dismutase) and in the cytosol that are able to counteract the excessive accumulation of reactive oxygen species (ROS) [5, 6]. It is known that physiological concentrations of ROS exert beneficial effects. On the other hand, increased oxidative stress, as a result of an imbalance between the production and the clearance of ROS, represents a relevant mechanism responsible of cell damage and death [7, 8]. In fact, proteins, lipids, and nucleic acids are the main target of excess ROS. Moreover, an increased level of inflammatory markers parallels that of oxidative stress, with a consequent state of chronic evolving pathological inflammation. At the organ level, increased ROS accumulation and inflammation are involved in the cardiovascular functional and structural damage underlying all major cardiovascular diseases (CVDs) [9].

Based on the key role that ROS play within the cardiovascular system and in all major CVDs, it would be important to monitor its level in human patients for both diagnostic and therapeutic purposes since changes in oxidative stress level parallel the progression of the pathological condition as well as the response to treatment. In this regard, a major limitation is obviously represented by the limited availability of tissue samples from both heart and blood vessels so that the use of circulating markers able to represent the condition within the cardiovascular system could overcome the problem. Notably, several studies performed over the last 15 years have identified the isolation of circulating leukocytes as a suitable method to represent systemic cardiovascular stress conditions with minimal invasive intervention. Since leukocytes circulate in the blood stream, they might reflect quite closely both systemic and cardiovascular metabolic state [10].

It is likely that this method, once adequately developed, may provide an eligible tool for both research and clinical purposes to examine the role of several oxidative stress-related mechanisms in the promotion and progression of CVDs.

The present review article is aimed at discussing the relevance of studies of circulating leukocytes in all major CVDs, from arterial hypertension to ischaemic heart disease and to the final stage of heart failure (Table 1). We also highlight the role of leukocytes as both bystander and actor in the context of CVDs.

## 2. Cellular Sources of ROS and of Antioxidants

Mitochondria represent the major source of ROS production inside the cells [4]. ROS are produced physiologically as by-products across mitochondrial complex I and complex III. This occurs during the forward electron transfer, whereas oxygen is partially reduced to form anion superoxide (O_2_^−^) [4]. The reverse electron transfer across electron transport chain complexes is another mechanism contributing to mitochondrial ROS production [4]. Nicotinamide adenine dinucleotide phosphate (NADPH) oxidases (NOXs) also represent an important source of ROS in the cardiovascular system [11, 12]. All members of NOXs (NOX1, NOX2, NOX4, and NOX5) generate O_2_^−^ by using NADPH as electron donor. Then, O_2_^−^ can be converted into hydrogen peroxide (H_2_O_2_) [11, 12]. NOXs are activated by different stimuli, such as cytokines, growth factor, and angiotensin II. NOX-derived ROS act as signaling molecules, regulating different cellular mechanisms, especially in the cardiovascular system [11, 12]. Other important cellular sources of ROS and reactive nitrogen species are those derived from xanthine oxidase and by the uncoupling of nitric oxide (NO) synthase [13, 14].

Different antioxidant mechanisms are able to counteract the excessive accumulation of ROS. These are expressed both within the mitochondrial compartment (uncoupling proteins, thioredoxins, glutathione peroxidase, and superoxide dismutase) and in the cytosol [5, 6]. Superoxide dismutases (SODs) represent an important defence against oxidative stress. SODs catalyze the dismutation of O_2_^−^ into H_2_O_2_ [15]. SOD2 is the isoform expressed in the mitochondria whereas SOD1 and SOD3 are expressed in the cytoplasm and in the extracellular compartment, respectively [15]. Mutations of SODs are associated with different diseases including CVDs [15]. Catalase, another enzyme able to reduce oxidative stress, is expressed in peroxisome, and it is devoted to the conversion of H_2_O_2_ into water and molecular oxygen (O_2_) [16]. Notably, catalase overexpression was found to reduce atherosclerosis in mice lacking apolipoprotein E [17].

## 3. Isolation of PBMCs and Detection of Oxidative Stress Level

The human PBMCs isolation procedure is based on widely optimized protocols of cell separation by gradient centrifugation. This method exploits the principle of differential migration of cells in specific density gradient media [18]. Usually, venous blood samples drawn into collection tubes containing EDTA are centrifuged in the presence of polysucrose medium (Ficoll). After centrifugation, the PBMCs layer, separated from the plasma, is collected and the cells are made available for further processing such as the establishment of primary cultures (e.g., for the development of specific experimental models) or the cryopreservation for long-term storage.

For the evaluation of intracellular oxidative stress, the PBMCs are incubated with chemiluminescent, bioluminescent, or fluorescent redox-active probes detecting cytoplasmic (e.g., 2′,7′-dichlorofluorescein diacetate or CellROX Oxidative Stress Reagent) or mitochondrial reactive species (MitoSOX Red mitochondrial superoxide indicator or HE, hydroethidine) [19, 20]. The signals exhibited by oxidized fluorescent probes can be assessed both in cell lysates and in whole cells by quantitative fluorescence microscopy and flow cytometry. This procedure offers some technical advantages when compared to the complex sample preparation processes required by the methods to detect protein carbonyls [21] or isoprostanes in plasma samples [22].

In addition, in experimental conditions where PBMCs primary cultures are available, the quantitation of reactive species metabolites, ROS scavengers, and antioxidant enzymes can be carried out by chromogenic and enzymatic assays from culture supernatants. Moreover, the gene expression analysis of PMBCs allows the evaluation of antioxidant systems and of other molecules involved in the modulation of intracellular oxidative stress, such as the OXPHOS genes [23–25]. Finally, the quantitative assessment of mitochondrial structure and function provide additional information when oxidative stress has a mitochondrial genesis [25–27].

## 4. Hypertension

The pathophysiology of hypertension is a complex and multifactorial process. Abnormalities of the sympathetic nervous system, of the renin-angiotensin-aldosterone system, of G protein-coupled receptor signaling, and of inflammatory and immunity mechanisms play a contributory role [28]. Of note, these processes lead to excess oxidative stress through increased ROS generation, decreased NO levels, and reduced antioxidant capacity in the cardiovascular system. Notably, the pathophysiological role that excess ROS plays in inflammation, hypertrophy, fibrosis, proliferation, migration, and angiogenesis becomes of key importance for the process of cardiovascular remodeling and ultimately of target organ damage development in hypertension.

Evidence that oxidative stress contributes to hypertension development comes from old studies [29–31]. However, only few investigations have been performed in the attempt to detect the level of ROS in hypertensive patients and their interaction with key parameters of the hypertensive disease. Interestingly, in a cohort of 529 hypertensive subjects, the level of polymorphonuclear leukocyte (PMN) oxidative stress significantly correlated with mean blood pressure (BP) level and also with hemoglobin A(1c) level. In the same cohort, a significant correlation was found between C-reactive protein level and mononuclear cell (MNC) oxidative stress [32]. Furthermore, subjects from this cohort affected by both hypertension and diabetes showed increased PMN and MNC oxidative stress. These findings, while documenting a tight relationship between hypertension and diabetes with oxidative stress and inflammation, had the merit to develop and propose the measurement of oxidative stress in circulating leukocytes as a valid tool to detect the hypertension-related vascular damage.

As a logical follow-up, the same authors investigated whether an increased level of oxidative stress may be detected in circulating leukocytes of extreme dipper and of morning BP surge-type hypertensives [33]. As expected, ROS formation by MNCs was significantly increased in both types of hypertensive patients. Furthermore, the combination of extreme dipper and of morning surge BP types led to an even higher level of ROS formation. These findings indicate that higher ROS formation from leukocytes in extreme dipper and morning surge BP types is a marker of predisposition to early morning cardiovascular events (CVE). It cannot be excluded that higher ROS level plays also a contributory pathogenic role into the development of CVE in these types of hypertensive patients. This hypothesis is strengthened by the observation that ROS formation by MNCs relates not only to nocturnal BP level but also to other cardiovascular risk factors, such as left ventricular mass, carotid intima-media thickening (IMT), and norepinephrine [34].

The usefulness of measurement of leukocyte oxidant activities may reveal importance not only for the evaluation of the oxidative stress in hypertension but also to monitor the effect of antihypertensive drugs. In this regard, it has been shown that the use of benidipine reduced oxidative stress in PMNs of hypertensive patients, at least in part by reducing BP levels [35], and that angiotensin II type 1 receptor (AT1R) blockers were able to normalize BP and to reduce the oxidative stress produced by leukocytes, thus suggesting that the latter plays an active role in the pathogenesis of hypertension through the production of oxidative stress. In fact, these studies proposed the leukocytes as a target of antihypertensive drugs [36–38].

## 5. Atherosclerosis and Ischaemic Heart Disease

Signals that regulate cellular proliferation, neointimal formation, and vessel wall thickening underlie the cellular and molecular basis of atherosclerotic plaque development. This process has both systemic oxidative stress and inflammatory components involving endothelial dysfunction, vascular smooth muscle cell proliferation and migration, circulating immune cells, and monocytes/macrophages [39, 40]. The latter has been shown to be involved in the development and progression of coronary artery disease (CAD) [41]. Moreover, it has been demonstrated that neutrophils play a key role in CAD progression and plaque instability through the generation of myeloperoxidase (MPO) [42]. In fact, the MPO deficiency reduced inflammation, oxidative stress, and plaque formation in the apolipoprotein E-deficient mice fed with high-cholesterol diet [42]. PMNs are tightly linked to other cells involved in the inflammatory responses at the vascular level, and they are modulated by lymphocytes and macrophages. Also, the disruption of mitochondrial function can elicit further oxidative stress. In fact, monocyte mitochondrial DNA damage and decreased complex I and IV activities have been identified in mouse models of atherosclerosis [43].

The use of peripheral blood leukocytes may represent a valid tool to determine the oxidative stress in patients affected by atherosclerosis and particularly by CAD. To support this concept, a study by Leu et al. demonstrated that a basal O_2_^−^ generation by MNCs was able to predict CVE in patients with cardiac syndrome X, independently from other risk factors. This observation suggested the potential role of measuring oxidative stress produced by MNCs both for monitoring CAD progression and for risk stratification in syndrome X patients [44]. However, this method has revealed no practical value in clinics so that other parameters of oxidative stress can be determined in circulating leukocytes.

### 5.1. Oxidative Stress-Related Gene Expression

Several studies have shown that the detection in PBMCs of the expression of genes involved in the atherosclerotic process reveals useful in terms of prediction and monitoring of the atherosclerotic vascular lesions. In a model of hypercholesterolemic rabbit, a significant relationship was observed between the aorta and PBMC CD36 mRNA expression [45]. The latter is a receptor that facilitates the uptake of oxLDL in the arterial wall [46–48]. It is expressed in intraplaque macrophages and mediates phagocytosis of oxLDL leading to foam cells formation [47]. More importantly, a significant correlation was found between PBMC CD36 mRNA expression and higher cholesterol level in humans [45, 49], thus indicating that the CD36 mRNA level of PBMCs could be used as a biomarker for the diagnosis of the atherosclerotic burden.

Consistently, it was reported that the expression of p66Shc, a mitochondrial protein driving the hyperglycemic cell damage, was higher in PBMCs of diabetic patients who lately developed macroangiopathy [50]. At the experimental level, it has been shown that p66Shc deletion prevents diabetic complications [51]. Thus, this marker can be proposed to predict the onset of vascular complications in diabetes.

The gene expression profiling in blood leukocytes has been proposed as a suitable approach to identify subjects at risk for CAD, although with several inconsistencies between different studies [24]. In fact, no definite information still exists on the genes truly predisposing to CAD development.

Apart from the assessment of CAD risk, the determination of gene expression in circulating leukocytes may help to identify changes in response to alterations of a physiological state. Therefore, we could characterize a specific pathological condition. In particular, we may be able to monitor the presence and progression of CAD as well as the effects of therapeutic interventions [24]. Very few studies have been conducted so far with the detection of oxidative stress-related gene expression level in PBMCs of CAD patients. In a study performed in an Italian cohort of patients affected by acute coronary syndrome (ACS), the PBMCs were used to test the gene expression of a mitochondrial complex I subunit (Ndufc2) and to assess the oxidative stress level dependent from mitochondrial dysfunction. In fact, NDUFC2 expression was significantly impaired, along with a significant reduction of the expression of few antioxidant genes and increased ROS level, whereas no relevant changes could be observed in PBMCs of patients affected by stable angina [25]. This study corroborated the role of increased mitochondrial oxidative stress in the pathogenesis of ACS by demonstrating the presence of mitochondrial dysfunction and of ultrastructural damage along with a relevant reduction of ATP level in PBMCs of the ACS patients. Notably, the mitochondrial complex I deficiency derived from NDUFC2 suppression is responsible at the vascular level of increased inflammation, apoptosis, and necrosis, of impaired endothelial integrity and angiogenesis, and of the release of markers of plaque instability [25]. To our knowledge, this is one of the best example supporting the usefulness of circulating PBMCs as a marker of mitochondrial oxidative stress that mirrors the vascular stress condition and is able to differentiate a state of ACS from that of stable chronic angina. Interestingly, it appears that PBMCs carry and possibly amplify within the bloodstream a mechanism, the mitochondrial dysfunction, which is directly involved in the pathogenesis of ACS. In fact, they might be considered not only as a bystander but also as an actor in the scene. From this point of view, it is even possible to identify PBMCs as a target for CAD treatment.

### 5.2. Telomere Length Assessment in PBMCs

An additional parameter that has been investigated in leukocytes is their telomere length. The leukocyte telomere length (LTL) is considered as a marker of biological aging [52–55]. In particular, oxidative stress plays a major role in the process of telomeric DNA loss [52–55]. In a study conducted in Chinese patients affected by premature CAD, the LTL was found to be shorter, and this phenomenon was associated with a reduced antioxidant capacity and increased ROS production [56].

An interesting study supports the use of circulating leukocytes to assess LTL as a marker of CAD. In particular, a relation was found between LTL and statin therapy in CAD patients: those under statin treatment had longer LTL as compared to patients without [57].

### 5.3. Proteomic Approach

It is clear that changes in several proteins involved in the oxidative stress response to heart ischaemia accompany the cardiac damage. In particular, since mitochondrial metabolism is heavily involved, changes in proteins of ETC are expected, particularly those of complex I proteins [58].

Their detection through a proteomic approach in PBMCs could help to detect and monitor the oxidative stress associated to CAD, to monitor the disease state itself and the response to treatment. However, the use of PBMC proteomics is still at its very early stage. Initial evidence of protein changes in PBMCs is emerging only with regard to human stroke [59].

Future studies using this approach in CAD are warranted.

## 6. Stroke

The pathogenesis of stroke, a common complex cardiovascular trait, is the result of several contributory factors including hypertension, genetics, and lifestyle. Also in the case of stroke, common mechanisms relate to vascular inflammation, proliferation, angiogenesis, apoptosis, and dysfunction. Experimental evidence highlights a key contributory role of increased oxidative stress in the development of stroke [60, 61].

In humans, we previously demonstrated that the rs11237379/NDUFC2 gene variant, associated to reduced mitochondrial complex I activity and increased oxidative stress, was a risk factor of juvenile ischaemic stroke [60]. Then, we showed that circulating leukocytes of healthy subjects carrying this gene variant have an altered mitochondrial function and relevant structural damage with increased mitochondrial oxidative stress in response to stress stimuli [26]. This interesting evidence underscores the potential role of circulating leukocytes as a marker of oxidative stress and of increased cardiovascular risk in genetically predisposed individuals.

Very few studies exploited the use of PBMCs to detect oxidative stress in the clinical context of cerebrovascular disease. In the study by Aizawa et al., the intracellular ROS level of PMNs was found to be higher in patients with ischaemic brain attack as compared to controls [62]. Notably, treatment with a free radical scavenger (edaravone) reduced the PMN ROS level as well as the O_2_^−^ production in these patients. Thus, this study revealed the importance of circulating mononuclear cells to monitor stroke and the response to a suitable therapy [62].

## 7. Heart Failure

Heart failure (HF) is a common clinical condition that represents the final stage of several cardiac diseases and of the underlying pathophysiological mechanisms. Among all, HF is characterized by excess oxidative stress, due to a relevant mitochondrial component, and by chronic inflammation. The myocardial oxidative stress deteriorates cardiac function by promoting cellular damage and death.

The PBMCs have been frequently used to detect mitochondrial respiratory dysfunction in HF, with evidence of a significant correlation with cardiac disturbances and clinical manifestations, already detectable at an early stage of the disease [63]. In fact, high levels of ROS and of O_2_^−^ are present in the blood of HF patients. Functional alterations of the antioxidant enzymes are detected in parallel in failing myocardium [64]. In addition, excess DNA double-strand breaks and higher level of DNA repair proteins are found in PBMCs of HF patients [65]. Moreover, the neutrophil O_2_^−^-generating capacity contributes to other deleterious phenotypes of HF such as endothelial dysfunction. The latter remained unchanged after short- and long-term therapy with vitamin C [66].

Of note, the spontaneous reaction of O_2_^−^ and NO generates the potent oxidant peroxynitrite. Protein nitration is a consequence of peroxynitrite production and is found to be higher in PBMCs of HF patients. In addition, the oxidative-nitrative stress leads to the activation of several enzymes, including matrix metalloproteinases and poly(ADP-ribose) polymerase-1 (PARP). The latter, once overactivated, consumes NAD+ and ATP, leading to cell apoptosis and death [67].

A recent study from our group has shown that PBMCs of patients with chronic HF produce excess ROS, have reduced mitochondrial functional performance, and present with ultrastructural damage such as disruption of the inner mitochondrial membrane (IMM) and low IMM/outer MM (OMM) index value [27]. These phenomena were paralleled by an impairment of the activation of antioxidant genes and by a reduced mitophagic flux, with a consequent inadequate mitochondrial dynamics. The functional and structural alterations observed in these patients are likely responsible of a further ETC impairment, mitochondrial uncoupling, and O_2_^−^ production.

Sirtuin 1 (SIRT1) is a factor involved in the cell survival mechanism, heart ischaemic injury, and the pathophysiology of CVDs [68]. SIRT1 expression in PBMCs was significantly reduced, particularly in decompensated HF, and it was significantly related to the increased oxidant level and decreased antioxidant capacity [69].

Therefore, all studies support the concept that dysfunctional circulating PBMCs can act as an amplifier of oxidative stress and of cell/tissue damage, ultimately contributing to the progression of the HF condition [70]. As a consequence, it is no surprise that PBMC ROS level predicts hospital readmission in chronic HF [71]. Moreover, as outlined above for hypertension and CAD, the PBMCs could become a target also for HF treatment.

The impact of therapies used in HF patients has been evaluated at the cellular level and in circulating leukocytes. Patients with decompensated chronic HF and marked systemic inflammation and increased production of oxygen free radicals improved their functional status and reduced their indices of inflammation and oxidative stress after short-term inotropic support. In particular, short-term inotropic support with milrinone and dobutamine improved clinical status, as assessed by NYHA classification and by the 6 min walk test, and significantly decreased plasma levels of inflammatory and oxidative stress markers at 30 days.

Since oxidative stress is amplified in HF because of absent pulmonary clearance of ROS-loaded circulating leukocytes and platelets, pulmonary removal of ROS and transfer of redox active molecules to the mitochondria can be proposed as targets for chronic HF therapy [70].

A study by Kong et al. evaluated the leukocyte mitochondrial alterations induced by bypass surgery and found that they are part of the systemic immune perturbations related to cardiac surgery [72]. The impact of implantation of the left ventricular assist device on the oxidative stress of circulating leukocytes has been also evaluated. Interestingly, after implantation of the left ventricular assist device (LVAD), an increased production of ROS was observed in the blood leukocytes indicating persistent oxidative stress in these patients [73]. Since the production of ROS and DNA damage relate to high shear stress, the most plausible explanation of the abovementioned phenomenon is that continuous flow LVAD generates high shear stresses, therefore favoring excess ROS production from circulating PBMCs and DNA damage from lymphocytes. Less traumatic devices are warranted to circumvent the problem.

## 8. Conclusions

A large body of evidence underscores the importance of monitoring oxidative stress in CVDs with the aim of achieving more information on the disease state and on the response to treatment. This observation implies that relevant prognostic information for the patients can be obtained in parallel. At the time being, no definite tool exists in the clinical practice. In particular, although some oxidative stress markers assessing the oxidation of phospholipid and LDL protein components predict increased CV risk, none of them have yet been incorporated into clinical practice [9]. In fact, clinical trials including cross-sectional and retrospective and prospective studies provided conflicting results [74], mostly due to methodological issues.

The choice to study the PBMCs redox state in the CVDs is specifically based on the evidence that these cells play a key role in the modulation of atherogenesis by orchestrating the inflammatory and immune responses [75]. Lymphocytes and monocytes have a tight interaction with intrinsic vascular cells in the atheroma. In this context, cytokines and metabolites of reactive species are the signals that mediate the relationships between different cell types. They also modulate many key processes such as the induction of endothelial dysfunction, the triggering of coagulation and fibrinolysis, the formation and accumulation of foam cells, the migration and reprogramming of vascular smooth muscle cells, and the activation repair pathways culminating in tissue fibrosis [76–78].

Furthermore, the redox state of PBMCs closely reflect the systemic cardiovascular oxidative stress condition and therefore the presence and progression of CVD. It is likely that an altered redox state of dysfunctional circulating leukocytes can act as an amplifier of oxidative stress at tissue level, worsening the evolution of the disease [63, 70, 79].

**Figure 1:**
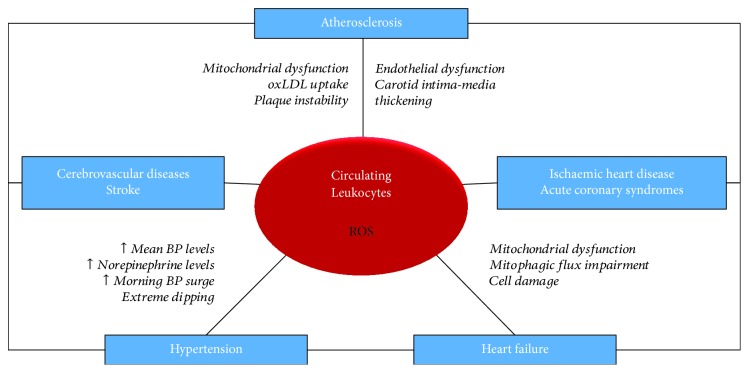
Circulating leukocytes and oxidative stress in cardiovascular diseases (CVDs). Schematic representation of the relationship between circulating leukocytes and reactive oxygen species (ROS) in different CVDs. The evidence collected in the context of the oxidative stress-induced cardiovascular damage points to a double role of leukocytes, both as a marker of disease condition and as an active player of disease progression.

Based on the evidence collected over the last few years and summarized in this article (Table 1, Figure 1), it is likely that the use of circulating leukocytes may become a relevant clinical tool. They may also serve to better understand the mechanisms involved in CVD pathogenesis. Nevertheless, more studies are needed to reinforce the current evidence and to identify the advantages, if any, of PBMCs compared to the markers detecting oxidative stress in plasma.

Finally, based on the evidence that PBMCs can directly distribute and amplify oxidative stress in the cardiovascular system, it is tempting to speculate that they may even be proposed as a target for the treatment of CVDs. However, since several difficulties would hamper this achievement in the clinical practice, the isolation of PBMCs to be used as a marker of oxidative stress level in CVDs remains the major and easier goal to pursue.

## Figures and Tables

**Table 1:** Relevant studies highlighting the correlation between the oxidative stress level detected in circulating leukocytes and the cardiovascular phenotypes/outcomes in CVDs.

Disease	Sample	Level of direct and indirect markers of oxidative stress	Cardiovascular phenotypes/outcomes	Reference
Hypertension	PMNs	↑ ROS	↑ BP	[32]

Hypertension	MNCs	↑ ROS	↑ C-reactive protein level	[32]

Hypertension	MNCs	↑ ROS	(i) Extreme dipper-type hypertension (ii) Morning BP surge-type hypertension	[33]

Hypertension	MNCs	↑ ROS	↑ Left ventricular mass, carotid IMT, nocturnal BP, norepinephrine level	[34]

Hypertension	PMNs	↓ ROS ↑ Antioxidant activity	↑ Response to antihypertensive agents ↓ BP	[35–38]

Cardiac syndrome X	MNCs	↑ ROS	↑ Cardiovascular events	[44]

Atherosclerosis	PBMCs	↑ CD36 expression	↑ Cholesterol level and oxLDL	[45]

Diabetes	PBMCs	↑ p66Shc expression	↑ Macroangiopathy	[50]

CAD	PBMCs	↑ ROS ↑ Mitochondrial dysfunction ↓ Antioxidant genes	ACS	[25]

CAD	PBMCs	↑ ROS ↓ Antioxidant defence ↓ LTL	Premature CAD	[56]

CAD	PBMCs	↑ LTL	Response to statin therapy	[57]

Stroke	PMNs	↑ ROS	↑ Ischaemic brain attack	[62]

HF	PBMCs	↑ ROS ↑ oxLDL ↑ DNA damage	↑ Severity of HF	[65]

HF	PBMCs	↑ ROS ↓ Antioxidant defence	SIRS development after CF-LVAD implant surgery	[73]

HF	PBMCs	↓ ROS	Response to vitamin C therapy Improved endothelial function	[66]

CHF	PBMCs	Mitochondrial dysfunction ↑ ROS ↓ Mitophagy	Not evaluated	[27]

HF	PBMCs	↓ SIRT1 ↑ ROS	Reduced cardiac compensation status	[69]

CHF	PBMCs	↑ ROS	↑ Hospital readmission	[71]

ACS: acute coronary syndrome; BP: blood pressure; CAD: coronary artery disease; HF: heart failure; CF-LVAD: continuous flow left ventricular assist device; CVDs: cardiovascular diseases; IMT: intima-media thickening; LTL: leukocyte telomere length; MNCs: mononuclear cells; oxLDL: oxidized low-density lipoprotein; PBMCs: peripheral blood mononuclear cells; PMNs: polymorphonuclear leukocytes; ROS: reactive oxygen species; SIRS: systemic inflammatory response syndrome; SIRT1: sirtuin 1.
